# Supplementary material for: Comparative genome analysis of Pseudogymnoascus spp. reveals primarily clonal evolution with small genome fragments exchanged between lineages
Source: BMC Genomics. 2015 May 21;16(1):400. doi: 10.1186/s12864-015-1570-9 (PMC4438637; doi:10.1186/s12864-015-1570-9)
Supplement: Additional file 1: Figure S1. — Distribution of VKM F-3808 genes by the number of P. spp. genome assemblies containing them. Figure S2. Phylogenetic relations between Pseudogymnoascus spp. VKM strains sequenced in this study and P. spp. strains from Minnis et al. [1] obtained from multuple sequene aligment of TEF1 gene. [file 12864_2015_1570_MOESM1_ESM.doc]

Comparative genome analysis of *Pseudogymnoascus spp.* reveals primarily clonal evolution with small genome fragments exchanged between lineages.

Evgeny V. Leushkin[1,2,*] leushkin@gmail.com, Maria D. Logacheva[1,2,3] maria.log@gmail.com, Alexey A. Penin[1,2,4] alekseypenin@gmail.com, Roman A. Sutormin[1,5] rsutormin@gmail.com, Evgeny S. Gerasimov[1,4] jalgard@gmail.com, Galina A. Kochkina[6] gak@dol.ru, Natalia E. Ivanushkina[6] nei@dol.ru, Oleg V. Vasilenko[6] ovvasilenko@gmail.com, Alexey S. Kondrashov[1,7] kondrash@umich.edu, Svetlana M. Ozerskaya[6] smovkm@gmail.com

[1]Department of Bioengineering and Bioinformatics, Lomonosov Moscow State University, Leninskye Gory 1-73, Moscow, 119992, Russia,

[2] Institute for Information Transmission Problems of the Russian Academy of Sciences, Moscow, 127994, Russia

[3]A.N. Belozersky Institute of Physico-Chemical Biology, Lomonosov Moscow State University, Moscow, Russia

[4]Department of Biology, Lomonosov Moscow State University, Moscow, 119992, Russia

[5]Lawrence Berkeley National Laboratory, Berkeley 94710, CA, USA.

[6]G.K.Skryabin Institute of Biochemistry and Physiology of Microorganisms RAS, Pushchino, 142290, Russia

[7]Department of Ecology and Evolutionary Biology, University of Michigan, Ann Arbor, MI 48109, USA

[*]Corresponding author

Running title: Population genomics of *Pseudogymnoascus spp.*`

Key words: asexual fungus, clonality, recombination, MAT-locus

Abstract

Background

Pseudogymnoascus spp. is a wide group of fungi lineages in the family Pseudorotiaceae including an aggressive pathogen of bats *P. destructans.* Although several lineages of *P. spp.* were shown to produce ascospores in culture, the vast majority of *P. spp.* demonstrates no evidence of sexual reproduction. *P. spp.* can tolerate a wide range of different temperatures and salinities and can survive even in permafrost layer. Adaptability of *P. spp.* to different environments is accompanied by extremely variable morphology and physiology.

Results

We sequenced genotypes of 14 strains of *P. spp.*, 5 of which were extracted from permafrost, 1 from a cryopeg, a layer of unfrozen ground in permafrost, and 8 from temperate surface environments. All sequenced genotypes are haploid. Nucleotide diversity among these genomes is very high, with a typical evolutionary distance at synonymous sites dS ≈ 0.5, suggesting that the last common ancestor of these strains lived >50Mya. The strains extracted from permafrost do not form a separate clade. Instead, each permafrost strain has close relatives from temperate environments.

We observed a strictly clonal population structure with no conflicting topologies for ~99% of genome sequences. However, there is a number of short (~100-10000 nt) genomic segments with the total length of 67.6 Kb which possess phylogenetic patterns strikingly different from the rest of the genome. The most remarkable case is a MAT-locus, which has 2 distinct alleles interspersed along the whole-genome phylogenetic tree.

Conclusions

Predominantly clonal structure of genome sequences is consistent with the observations that sexual reproduction is rare in *P. spp*. Small number of regions with noncanonical phylogenies seem to arise due to some recombination events between derived lineages of *P. spp.*, with MAT-

locus being transferred on multiple occasions. All sequenced strains have heterothallic configuration of MAT-locus.

Introduction

Pseudogymnoascus spp. is a group of fungi species which phylogenetically belongs to the phylum *Ascomycota,* family *Pseudeurotiaceae*. Many of the *P. spp.* including *P. destructants* were known as *Geomyces spp.* until reclassification based on phylogenetic analysis conducted in [1]. Species boundaries in *Pseudogymnoascus* still remain uncertain [1] recalling an overall problem in fungal taxonomy [2]. *P. spp* were long time believed to be anamorphic based on the absence of the evidence sexual reproduction [3–6], *P. destructants* was shown to spread clonaly in North America [7]. However, several homothallic lineages of *P. spp* were shown to produce ascospores in culture [1, 8], while *P. destructants* was proposed to have a heterothallic sexual reproduction pathway.

Morphology of *P. spp.* varies dramatically dependent on the growing conditions [9]. *P. spp.* are widespread in soils and can be found almost everywhere from Arctica to Antarctica [10]. *P. spp.* can tolerate low temperatures and high salinity, although they are not truly psychrophilic or halophilic [11–14]. *P. spp.* can degrade keratin and cause skin infections [15], and *P. destructans* causes white nose syndrome in bats [16].

Strictly asexual reproduction should result in clonal structure of population. However, sex is often hard to detect in experimental studies on *Ascyomycota* species [17]. Also many *Ascomycota* species are capable of parasexual process, which consists of fusion of cells followed by chromosome loss which eventually restores the normal caryotype, but does not involve meiosis. Parasexual process is often accompanied by recombination, although its rate is lower than that of meiotic recombination and it affects only short chromosome segments [18, 19].

Horizontal gene transfer (HGT) can also occur in fungi. The most common type of HGT involves homologous recombination between genome sequences [20]. Although most of the cases reported so far involve HGT between different species [21], one can expect that within-population HGT which involves homologous recombination is even more common [20, 22]. Thus, even if *P. spp.* truly lack meiosis, there still could be some genetic exchanges between strains in its populations.

Whole-genome analysis of *P. spp.* enables us to investigate such recombination events and detect genes associated with recombination activity. It also reveals relation between strains extracted from permafrost and temperate environments, which are considered isolated. Here, we report data on the genetic structure of *P. spp.* strains.

Results

Genome assembly, annotation, and key characteristics of *P. spp.* genomes

We performed whole-genome sequencing and analysis of 14 *P. spp.* strains.
These strains were collected from different habitats: temperate environment and Arctic active layers (contemporary samples), permafrost (age is 1.8-3.0 myr) and cryopeg, a layer of unfrozen ground in permafrost, (age is 120,000-200,000 years), and from different geographic locations (Table 1). None of the strains was seen to produce ascospores. Sequencing was performed on HiSeq2000 machine using paired-end libraries with average insert size ~350 nt. The sequenced reads were assembled, independently for each individual, with SOAPdenovo (v. 1.05). Assembly statistics for each strains are listed in Table 2. Whole-genome alignments of the assembled genotypes was created with LASTZ and CLUSTAL (see Materials and Methods). Mapping reads to their assembly reveals that all studied *P. spp.* isolates are haploid.

Annotation of genomes of the sequenced strains was performed with Augustus [23] v.2.7. Number of annotated genes within a genome varies from 9516 to 12470 (Table 3). The vast majority of genes is present in all or almost all assemblies (Fig. S1), e.g. out of 11305 genes in strain VKM F-3808, 8495 genes were identified in at least 10 other assemblies and 487 were not found in any other assembly. Using CEGMA pipeline [24], we demonstrated that for all *P. spp.* strains except F-3775, ≥90% of low-copy Core Eukaryotic Genes are fully present in the assembly (Table 3). Considerable variation of the number of annotated genes among genomes could be due to difference in assembly quality. However, separate analysis of genes pseudogenized or deleted on specific branches of the phylogenetic tree indicates asymmetric loss of genes among *P. spp.* strains (Fig. 1A). Strains F-4281, F-4246, and F-4513 have the lowest numbers of genes and the highest rates of gene loss ($1.0–2.4×10^{-5}$ per silent nucleotide substitution), whereas strains F-4518 and F-4520 have the highest number of genes and the lowest rates of gene loss ($1.4–1.5×10^{-6}$ per silent nucleotide substitution) (Fig.1A). Overall we detected 282 lost genes (145 deleted and 137 pseudogenes).

The GC-content varies from 49.1% to 51.1% (Table 3) among the sequenced strains, with the average 50.3%. More than half of the genome consists of protein-coding genes. The total length of genes varies from 16.4Mb to 21.8 Mb among the strains, and the total length of intergenic regions varies within a wider range from 7.0Mb to12.8Mb. Average gene lengths are 1438–1828bp, average numbers of introns per gene are 1.75–2.48, average intron lengths are 102–111bp, median intron length is 58–60bp (Table 3).

We also compared sequences obtained in our study to sequences of *P. pannorum* obtained previously in other studies. Genotype sequence of strain F-4281 is very similar (id = 99%) to genotype sequence of *P. spp.* strain sequenced in [25]. We also combined our data with [1] (based on ITS region, LSU, MCM7, RPB2, and TEF1) and attributed our strains to different

clades of *P. spp.* obtained in that study (Fig. S2). Our strains correspond to 7 different clades of *P. spp.* from [25].

Relationships between 14 *P. pannorum* genotypes

Comparison of the genomes of *P. spp.* strains reveals their very high nucleotide diversity. A typical genetic distance between two sequences at synonymous sites, dS, is ~0.5, although some strains form compact clades (Fig. 1A) and are much closer to each other. For strains from different clades, a typical distance at nonsynonymous sites dN is ~0.04 (Fig. 1B). Synteny between all genomes is extensive, and even within the most distant genome pairs over 90% of orthologous gene pairs are followed by another pair of orthologous genes (Fig. 2, Table S1, see also Materials and methods). There are no traces of either geographical or geological structure of the global population of *P. spp.* in the phylogenetic relationships among the analyzed genomes. Thus, these structures, if they exist, must be much younger than divergence of the ancestral lineages of these genomes.

Topology of the phylogeny shown on Fig. 1 holds throughout almost the entire genome. Only 0.47%, 0.31%, 0.05%, 1.27%, and 0.58% of whole genome alignments do not support the 5 clades, (VKM F-3808, VKM F-3557, VKM F-4514, VKM F-4516), (VKM F-4246, VKM F-4513), (VKM F-4515, VKM F-4517), (VKM F-103, VKM F-4519), and (VKM F-4518, VKM F-4520), respectively (Table 4). This implies that regular recombination does not take place between the *P. spp.* strains and supports the observations of primarily asexual reproduction in *P. spp.* Clade (VKM F-3808, VKM F-3557, VKM F-4514, VKM F-4516), the only clade with more than two strains, demonstrates a strong linkage disequilibrium among genotypes from the same clade (Fig. 3A). No linkage disequilibrium was observed at distances over 20 nucleotides for genotypes from different clades (even at nonsynonymous sites) (Fig. 3BC), which is likely due to homoplasy between highly diverged (dS~0.5) sequences and little time intervals between

lineage splits. Strains VKM F-3557, VKM F-4515, VKM F-4246 were used to demonstrate relations between distant clades, however the results are similar to that observed on Fig. 3BC for any combination of distant strains.

Search for meiotic genes and mating pathway genes

We searched for the genes orthologous to those which are responsible for meiosis or mating in *S. cerevisiae.* In *P. spp.* genomes we found orthologs for 17 out of 31 genes involved in different steps of meiosis in *S. cerevisiae* (Table 5), implying that 14 out of these 31 genes were lost in *P. spp.*. 11 out of 14 lost genes are involved in early phases of meiosis in *S. cerevisiae*: ime1 and rec12 are meiosis-inducing protein [26, 27], mum2 is required for premeiotic DNA synthesis [28], red1 is required for segregation of chromosomes in meiosis I [29], zip1, zip2, zip3 and zip4 are required for initiation of chromosome synapsis [30, 31]; the rest 3 of these genes, dit1, isc10 and mum3 are involved in sporulation in *S. cerevisiae* [32, 33] .

In contrast to meiotic genes we observed only 1 lost gene out of 21 which are responsible for mating in *S. cerevisiae* (Table 5), notably all STE genes responsible for mating factor sensitivity in *S. cerevisiae* are also present in *P. pannorum* strains [34]. A putative mating-type (MAT) locus with highly-conserved apn2 and sla2 genes was also found in *P. spp.* (Fig. 4, Table S2). We sequenced MAT-locus in 16 additional strains of *P. spp.* to study it in more detail. Two distinct idiomorphs of MAT-locus were observed: MAT1 idiomorph includes homolog of *MAT1-1-1* α-box transcription factor, homolog of *MAT1-1-3* high-mobility group (HMG) transcription factor, and an unknown gene which corresponds to *MAT1-1-6* in [8] (Fig. 4A); MAT2 idiomorph includes *MAT1-2-1* HMG-box gene and an unknown gene which corresponds to *MAT1-2-5* in [8] (Fig. 4B). Phylogenetic configuration at MAT-locus (Fig. 5A) is strikingly different from the rest of the genome (Fig.1, Fig. 5B). The boundaries of the segment with altered phylogeny reside at the ends of *MAT1-1-3* and *MAT1-1-1* genes for MAT1 idiomorph

and *MAT1-2-1* and *MAT1-2-5* for MAT2 idiomorph, so that flanking regions have canonical phylogenetic configuration (Fig. 5B). The last ~150 nucleotides of *MAT1-1-1* and *MAT1-2-5* are homologous to each other and unlike the rest of MAT-locus have canonical phylogenetic configuration. Multiple clades with both variants of MAT-locus and slightly variable boundaries of such segments in different strains indicate multiple recombination events within the MAT-locus (Fig. 5).

No paralogs of MAT-locus were found across *P. spp.* genomes, indicating that the observed pattern could not arise due to intragenomic conversion and, instead, implying multiple intergenomic recombination events at MAT-locus. Analysis of the MAT-locus indicates that all sequenced strains are heterothallic. *P. spp.* strain sequenced by [25] and *P. destructants* sequenced by "*Geomyces destructans* Sequencing Project" (http://www.broadinstitute.org/) also heterothallic and both have MAT1 configuration. According to [8] homothallic configuration with two idiomorphs combined also occurs in *P. spp.*, however no homothallic strain was detected among 14 fully-sequenced strains and 16 strains with only MAT-locus sequenced suggesting that homothallism is rare in *P.spp*.

Analysis of genomic regions with altered phylogenies

Genotypes of VKM F-3808, VKM F-3557, and VKM F-4514 form a tight clade (all other clades have 2 or 1 genotypes) and can be used to estimate the impact of recombination on *P. spp.* population in more detail. For this clade we performed whole-genome search for the regions with altered phylogenetic configuration. Within the alignment of VKM F-3557, and VKM F-4514 genotypes to the rest of 12 *P. spp.* genotypes, there are 77 relatively short regions, of the total length of 67.6 Kb and average length of 878nt (Fig. 6A, Table S3), where phylogenetic relationships between genotypes VKM F-3808, VKM F-3557, and VKM F-4514 differ significantly (Kishino-Hasegawa test) from their canonical topology (VKM F-3808, (VKM F-

3557, VKM F-4514)) (see Materials and Methods for the details of identifying these regions). Among these regions, 34 supported topology (VKM F-3557, (VKM F-3808, VKM F-4514)) and 43 supported topology (VKM F-4514, (VKM F-3808, VKM F-3557)) (Table S3). Average nucleotide divergence between VKM F-3557 and VKM F-4514 in such regions is 0.115 compared to genome average 0.015 (Fig. 6B, Table S3).

Fig. 7 describes one of such regions. VKM F-4514 becomes an outgroup to VKM F-3557 and VKM F-3808 inside the recombined region (Fig. 7B) in contrast to the flanking regions which maintain the canonical phylogenetic configuration (Fig. 7AC). The genetic distances from recombined strain to strains from outside clades are not increased in this example as well as in the other regions with noncanonical phylogenetic configuration (see F-4515 *vs.* F-3557 and F-4515 *vs.* F-4514 in Table S3) . Thus, such regions did not arise due to hypermutation and, instead, were likely generated through some sort of the recombination events. In one case (Fig. 8), a genomic region which supported (VKM F-3557, (VKM F-3808, VKM F-4514)) topology was marked by a 5.3kb inversion present in VKM F-3808 and VKM F-4514 genotypes but not in any other *P. spp.* genotypes. This inversion was preceded by ~100 nt non-inverted segment which also supported (VKM F-3557, (VKM F-3808, VKM F-4514)) topology. Such a complex situation is very unlikely to arise through independent reversing mutations.

Sequence reads mapped back to assemblies ensure that regions with altered phylogenetic topologies could not be assembly artifacts as reads map normally on such regions and on their boundaries, with average coverage for this regions being the same to the rest of the genome. We considered a possibility of the intragenomic nonallelelic recombination. For 3 of the 77 regions we identified paralogs inside *P. spp.* using BLAST against the entire genome. However, none of these 3 paralogs could explain the pattern we observed.

The most plausible explanation for the regions with altered topology is the weak recombination activity between the distant *P. spp.* lineages. In the first example (Fig. 7), VKM F-4514 likely recombined with some genotype outside of (VKM F-3808, (VKM F-3557, VKM F-4514)) clade, in the second example the inversion took place before the (VKM F-3808, (VKM F-3557, VKM F-4514)) branching, but was eventually eliminated in VKM F-3557 by recombination with some distant genotype (Fig. 8).

Exon sequences comprise 50.1% of the *P. spp.* genome, but only 11,345 nt in 23 regions out of the total 67,577 nt in 77 recombination regions (16.8%) overlap with exon sequences. The lack of coding sequences in recombination regions is likely due to a negative selection on high-distant recombination events at coding sequences.

Discussion and conclusions

We sequenced and independently assembled genotypes of 14 haploid *P. spp.* strains. Thus, we did not perform standard genotyping procedures including read mapping and SNP calling but, instead, aligned the contigs which were obtained independently. We believe this method to be preferable to read mapping, because longer sequences are aligned and more robust alignments are obtained. Longer sequences are particularly important in case of high nucleotide diversity within the aligned genotypes.

Genome comparison of the sequenced strains reveals predominantly clonal structure of *P. spp.* lineages (Fig. 3A, Table 4) which is consistent with the multiple observations of asexual-only reproduction of *P. spp.* strains [1, 3–6]. The sequenced genomes are also very diverse with typical distance between strains from different clades $dS \approx 0.5$. Assuming that *P. spp.* produce no more than 10 generation per year [9] and mutation rate is less than 10^{-8} per nucleotide per generation (similar to other *S. cerevisiae* [35, 36]), we can estimate that the last common

ancestor of *P. spp.* lived more than 50 Mya. However, the strains are still very similar in functional sites as dN between distant lineages is ~0.04, synteny of genes is >0.9 between different clades (Fig. 2).

Complete absence of genetic exchanges between strains would lead to a strict clonality of the population. However, there are evidences of recombination within a number of genes in anamorphic *Candida albicans* and *Aspergillus fumigatus* [37, 38]. We also observed such evidence in *P. spp.*. Phylogenetic structure of MAT-locus and other regions with noncanonical topology indicates the exchange of this genome fragments between *P. spp.* lineages (Figs. 4-8, Table S3). Recombination rate estimated from these regions is low: 1 recombination event per ~2500 single-nucleotide substitutions at synonymous sites, and only short genome regions are affected (average length is 878nt) (Fig. 6). However, it is enough to cover an entire genome for a period of time passed since last common ancestor of *P. spp.* lineages, and thus, also contributed to the loss of linkage disequilibrium (Fig.3).

As in an asexual fungi *Candida glabarta* [39, 40], in *P. spp.* we observed MAT locus and other genes responsible for mating and meiosis in *S. cerevisiae* (Table 5, S2, Fig. 4). Interestingly, MAT locus in *P. spp.* has phylogenetic configuration very different from the rest of the genome, indicating multiple transmissions between distant lineages at MAT locus. However, in both *Candida glabarta* and in vast majority of *P. spp.* sexual reproduction has never been observed, suggesting that either sexual processes are extremely rare, and thus are hard to detect, but are still important in these species, or that these genes have some functions other than sexual reproduction. Evidence of recombination at MAT locus and in other genome regions could also indicate parasexual activity which is known to be a substitute of sex for many *Ascomycota* [19]. The other explanation could be horizontal gene transfer (HGT) across *P. pannorum*. HGT better

fits the pattern observed for MAT-locus phylogeny and could indicate presence of a vector which carries and transmits MAT-locus across the population.

There are many economically significant species among Ascomycota, including aggressive pathogens of plants and animals. Recently *P. destructans* was shown to spread rapidly in North America and decimate bats populations [7, 16]. However, population genetics and evolution of Ascomycota species remain poorly understood due to low number of whole-genome data. Our analysis reveals predominantly clonal evolution of *P. spp.* lineages. But despite a very long time passed since their last common ancestor, these strains still have very similar morphological traits and evidently occupy the same ecological niche. Indeed, strains VKM F-4513, VKM F-4514 and VKM F-4517, which belong to 3 distant clades (Fig. 1), were all extracted from the permafrost samples of the same age (1.8-3.0 Myr) where no other organism could survive. Furthermore, sequenced genomes indicate some sort of genetic recombination between diverged lineages. Therefore we believe that *P. spp.* should be treated as the entity of lineages interacting with each other rather than an ensemble of independent species. This approach could also be useful in understanding evolution of the other Ascomycota species with little or unknown sexual reproduction.

Materials and methods

Extraction and cultivation of samples from permafrost

Methods of sampling, storage, transportation and control were chosen, and specialized tests were performed, to make sure that the microorganisms found in samples were indigenous and not contaminants. The cores (diameter 5-10 cm, length 15-30 cm) were collected using a dry drilling technique developed specifically for microbiological studies of permafrost [41, 42]. The dry drilling and sampling prevent down-hole contamination caused by drilling fluids. The sampling is achieved by dry shaving of the core back to native ice-cemented sediment. Possible

contamination during the drilling was monitored by several tests. Previous studies have employed fluidless drilling techniques combined with an exogenous bacterial tracer such as a pure culture of *Serratia marcescens*. In tests using the isolation techniques, *S. marcescens* bacteria were found only on the surface of the frozen sample, never inside the frozen cores [42].

To recover fungi, 0.5-g portions of a core sample were placed in test tubes with 5 ml of water heated to room temperature (20°C), as well as to 35 and 52°C. Following one minute, the suspension was shaken at room temperature for 10 min. The tenfold dilutions of this suspension were inoculated, in triplicate, on Czapek agar (Cz), Malt Agar (MA), Starch ammonium agar (SAA) to which lactic acid was added at a concentration of 4 ml/1 to suppress the unwanted growth of bacterial cells. The inoculated plates were incubated at 4 and 25°C. The grown colonies were examined and enumerated on the 21st and 30th days, respectively [43].

Genome sequencing

Before DNA extraction, all samples were grown on Malt Agar for 10 days. Total genomic DNA was extracted using modified CTAB-method [44]. To construct the libraries for whole genome sequencing DNA was processed as described in the TruSeq DNA Sample Preparation Guide (Illumina). Libraries with average length of 350 bp were selected for sequencing. Libraries were quantified using fluorimetry with Qubit (Invitrogen, USA) and real-time PCR and diluted up to final concentration of 8 pM. Diluted libraries were clustered on a paired-end flowcell using cBot instrument and sequenced in 101 cycles using HiSeq2000 sequencer with TruSeq SBS Kit v3-HS (Illumina, USA). After trimming of adapter-derived and low (Q-score below 30) quality sequences reads were assembled using SOAP de novo assembler application (k-mer size 57). GapCloser for SOAP de novo was used to determine sequences of the gaps in scaffolds [45].

RNA sequencing

RNA-seq was performed for strains F-3808 and F-4515 grown in control conditions (malt agar, temperature 25 C) and under low temperature and high salinity (). Prior to RNA extraction, samples were collected in RNAlater solution (Ambion, USA), then homogenized using liquid nitrogen. Extraction was carried out using RNeasy Mini Kit (Qiagen, Germany) following manufacturer's instruction. The only modification was the addition of 10% Plant RNA Isolation Aid (Ambion, USA) to the lysis buffer. RNA quality was assessed using capillary electrophoresis on Bioanalyzer 2100 (Agilent, USA), only RNA with integrity number (RIN, [46]) greater or equal to 8 were taken for library preparation. For library preparation, TruSeq RNA Sample Prep Kit v2 (Illumina, USA) was used following manufacturer's instructions. After preparation libraries were quantified using Qubit fluorometer and quantitative PCR and sequenced on HiSeq2000 with read length 51 nucleotide.

MAT locus sequencing

MAT locus was amplified using primers Geo-MAT1-2-F (5'-ATG GCT CAA AGC ACR TTG CAR GGC TTC-3') and Geo-MAT1-2-R (5'-CTT CTT TAT CTG GAC GTC ACT TCT CAC A-3') that encompass the region between genes *sla2* and *apn2*. PCR products were run on agarose gel and bands between 3 and 9 Kb were cut and purified. Libraries were prepared using Nextera XT DNA sample prep kit (Illumina, USA) and sequenced using Miseq sequencer with read length equal to 250 from each end. Libraries were 200-800 bp in length.

Genome annotation

Gene predictions for 14 *P. spp.* strains were done as described further. Each genotype assembly file was masked using RepeatMasker 3.3.0. To find exons and intronsRNAseq data we had for strains F-3808 and F-4515 were mapped on the masked scaffolds of each strain using Tophat2 [47] (version 2.0.8) and the results were used to generate intron hints for AUGUSTUS gene

predictor (with bam2hits and filterBam programs from AUGUSTUS pipeline, included in distributive, and samtools package for sorting and filtering). AUGUSTUS extrinsic.cfg file was adjusted for considering information about potential intron boundaries from RNAseq data (larger bonus for intron confirmed by RNA mapping, tiny penalty if not).Final gene prediction was done by AUGUSTUS [23] (version 2.7.) with intron hints and species parameter was set to "botrytis_cinerea".

Whole genome alignment

Whole-genome alignment of the assembled contigs was performed in 2 steps. First, we used LASTZ [46], the program which identifies the regions of local similarity, to match the contigs from different samples. Single_cov2 from TBA package [47] was used to filter out the lower-scored alignments in regions with more than one significant alignment. Then, to increase the length of the alignment blocks, we performed global alignment of contig groups obtained on stage 1 using CLUSTAL. For the analysis of the genomic regions with the conflicting phylogenetic configuration we only used the alignment blocks of length >20kbp. The entire length of such blocks is 5.6Mbp.

Identifying regions with noncanonical phylogeny

We considered a nucleotide site to support phylogenetic configuration (strain A , (strain B, strain C)), if nucleotides in strain B and strain C are identical and distinct from nucleotide in strain A, also we required nucleotide in strain A to be carried by at least 6 of the rest 11 sequenced *G. pannorum* strains. Phylogenetic configuration (VKM F-3808, (VKM F-3557, VKM F-4514)) was name canonical as it stands for the vast majority of the genome, whereas phylogenetic configuration (VKM F-3557, (VKM F-3808, VKM F-4514)) and (VKM F-4514, (VKM F-3808, VKM F-3557) were named non-canonical. The nucleotide frequency of sites with noncanonical phylogenetic configuration is 0.002.

We considered a window of length 200 nt to have a noncanonical phylogenetic configuration, if the number of nucleotide sites supporting a noncanonical phylogenetic configuration exceeds the number of sites with canonical phylogenetic configuration by at least 8 nucleotides. The threshold of 8 guaranties that less than 0.01 such windows would be found at random. The overlapping windows were combined into the resulting regions with the boundaries set at nucleotide sites supporting noncanonical phylogenetic configuration. PAML implementation of Kishino-Hasegawa test was run to compare phylogenetic configurations and calculate bootstrap values[49], pRELL threshold was set at 0.95.

To ensure the regions with altered phylogenetic configuration are not assembly artifacts, we mapped the original sequence reads using bwa [48] program on the regions with noncanonical phylogenetic configuration, overlapping the boundaries of the region to ensure that these region are not the assembly artifacts. Regions with noncanonical phylogenetic configuration show coverage similar to the rest of the genome.

Calculating phylogenetic distances, number of gene losses and synteny

To identify gene orthologs we searched bidirectional best hits for each pair of *P. spp.* strains. We obtained 7524 groups of homologous genes, which are present in each of these 14 strains. Then, each group of homologous genes was aligned with MACSE [49]. Finally, the concatenate of alignments was used to calculate synonymous and nonsynonymous distances with codeml program from PAML-package. Only codon columns present in all 14 strains were used in the analysis. Dendroscope (v. 3.2.10) was used for visualizations of phylogenies [52]. We evaluate number of genes lost on each branch from sets of orthologs which have no blast hits to exon sequences in certain lineages. The lost gene is considered pseudogene if the significant blast hit

to genome is observed but gene structure is disrupted, the gene is considered deleted if there is no significant blast hit to genome .

Gene orthologs were also used to estimate synteny across *P. spp.* strains. The pair of two orthologous genes was considered syntenic if those genes were adjacent in each strain. The pair of two orthologous genes where genes were adjacent only in one strain was considered nonsyntenic. Total numbers of syntenic orthologous pairs out of all orthologous pairs are shown in Table S1.

Data access

Raw sequence reads, genotypes assembly and annotation for 14 *P. spp.* strains are available in the Genbank under BioProject accession number PRJNA216963.

Competing interests

Authors declare that they have no competing interests.

Authors' contribution

EVL carried out computational analysis, coordinated the study and prepared the manuscript, MDL participated in construction and sequencing of DNA libraries, participated in coordination of the study and contributed to manuscript preparation, AAP participated in construction and sequencing of DNA libraries, RAS and ESG participated in genome annotation, GAK, NEI and OVV participated in extraction and cultivation of samples, ASK participated in design and coordination of the study and contributed to manuscript preparation, SMO conceived the study, participated in extraction and cultivation of samples and manuscript preparation.

Acknowledgments

This study was supported by Ministry of Education and Science of the Russian Federation grants №11.G34.31.0008 and 8814, by the Russian Foundation for Basic Research (grant no. 13-04-02082A), and by the Molecular and Cellular Biology Program of the Russian Academy of Sciences.

References

1. Minnis AM, Lindner DL: Phylogenetic evaluation of Geomyces and allies reveals no close relatives of Pseudogymnoascus destructans, comb. nov., in bat hibernacula of eastern North America. *Fungal Biol* 2013, 117:638–649.

2. Hibbett DS, Taylor JW: Fungal systematics: is a new age of enlightenment at hand?. *Nat Rev Microbiol* 2013, 11:129–133.

3. Hoog GSD: *Atlas of Clinical Fungi, Second Edition*. Amer Society for Microbiology; 2000.

4. Sigler L, Lumley TC, Currah RS: New species and records of saprophytic ascomycetes (Myxotrichaceae) from decaying logs in the boreal forest. *Mycoscience* 2000, 41:495–502.

5. Kirk PM, Cannon PF, Minter DW, Stalpers JA: *Dictionary of the Fungi*. 2008.

6. Hayes MA: The Geomyces Fungi: Ecology and Distribution. *BioScience* 2012, 62:819–823.

7. Ren P, Haman KH, Last LA, Rajkumar SS, Keel MK, Chaturvedi V: Clonal Spread of Geomyces destructans among Bats, Midwestern and Southern United States. *Emerg Infect Dis* 2012, 18:883–885.

8. Palmer JM, Kubatova A, Novakova A, Minnis AM, Kolarik M, Lindner DL: Molecular characterization of a heterothallic mating system in Pseudogymnoascus destructans, the Fungus causing white-nose syndrome of bats. *G3 Bethesda Md* 2014, 4:1755–1763.

9. Kochkina GA, Ivanushkina NE, Akimov VN, Gilichinskiĭ DA, Ozerskaia SM: [Halo- and psychrotolerant Geomyces fungi from arctic cryopegs and marine deposits]. *Mikrobiologiia* 2007, 76:39–47.

10. Marshall: Aerial Transport of Keratinaceous Substrate and Distribution of the Fungus Geomyces pannorum in Antarctic Soils. *Microb Ecol* 1998, 36:212–219.

11. Poole NJ, Price PC: The occurrence of Chrysosporium pannorum in soils receiving incremental cellulose. *Soil Biol Biochem* 1971, 3:161–166.

12. Lowry PD, Gill CO: Temperature and water activity minima for growth of spoilage moulds from meat. *J Appl Bacteriol* 1984, 56:193–199.

13. Robinson CH: Cold adaptation in Arctic and Antarctic fungi. *New Phytol* 2001, 151:341–353.

14. Ozerskaya S m., Ivanushkina N e., Kochkina G a., Fattakhova R n., Gilichinsky D a.: Mycelial fungi in cryopegs. *Int J Astrobiol* 2004, 3:327–331.

15. Gianni C, Caretta G, Romano C: Skin infection due to Geomyces pannorum var. pannorum. *Mycoses* 2003, 46:430–432.

16. Gargas A, Trest MT, Christensen M, Volk TJ, Blehert DS: Geomyces destructans sp. nov. associated with bat white-nose syndrome. *Mycotaxon* 2009, 108:147–154.

17. Ni M, Feretzaki M, Sun S, Wang X, Heitman J: Sex in Fungi. *Annu Rev Genet* 2011, 45:405–430.

18. Bennett RJ, Johnson AD: Completion of a parasexual cycle in Candida albicans by induced chromosome loss in tetraploid strains. *EMBO J* 2003, 22:2505–2515.

19. Forche A, Alby K, Schaefer D, Johnson AD, Berman J, Bennett RJ: The parasexual cycle in Candida albicans provides an alternative pathway to meiosis for the formation of recombinant strains. *PLoS Biol* 2008, 6:e110.

20. Mau B, Glasner JD, Darling AE, Perna NT: Genome-wide detection and analysis of homologous recombination among sequenced strains of Escherichia coli. *Genome Biol* 2006, 7:R44.

21. Fitzpatrick DA: Horizontal gene transfer in fungi. *FEMS Microbiol Lett* 2012, 329:1–8.

22. Chan CX, Beiko RG, Darling AE, Ragan MA: Lateral transfer of genes and gene fragments in prokaryotes. *Genome Biol Evol* 2009, 1:429–438.

23. Stanke M, Diekhans M, Baertsch R, Haussler D: Using native and syntenically mapped cDNA alignments to improve de novo gene finding. *Bioinformatics* 2008, 24:637–644.

24. Parra G, Bradnam K, Ning Z, Keane T, Korf I: Assessing the gene space in draft genomes. *Nucleic Acids Res* 2009, 37:289–297.

25. Chibucos MC, Crabtree J, Nagaraj S, Chaturvedi S, Chaturvedi V: Draft Genome Sequences of Human Pathogenic Fungus Geomyces pannorum Sensu Lato and Bat White Nose Syndrome Pathogen Geomyces (Pseudogymnoascus) destructans. *Genome Announc* 2013, 1:e01045–13.

26. Lin Y, Smith GR: Transient, meiosis-induced expression of the rec6 and rec12 genes of Schizosaccharomyces pombe. *Genetics* 1994, 136:769–779.

27. Kassir Y, Granot D, Simchen G: IME1, a positive regulator gene of meiosis in S. cerevisiae. *Cell* 1988, 52:853–862.

28. Davis L, Barbera M, McDonnell A, McIntyre K, Sternglanz R, Jin Q, Loidl J, Engebrecht J: The Saccharomyces cerevisiae MUM2 gene interacts with the DNA replication machinery and is required for meiotic levels of double strand breaks. *Genetics* 2001, 157:1179–1189.

29. Thompson EA, Roeder GS: Expression and DNA sequence of RED1, a gene required for meiosis I chromosome segregation in yeast. *Mol Gen Genet MGG* 1989, 218:293–301.

30. Chua PR, Roeder GS: Zip2, a meiosis-specific protein required for the initiation of chromosome synapsis. *Cell* 1998, 93:349–359.

31. Agarwal S, Roeder GS: Zip3 provides a link between recombination enzymes and synaptonemal complex proteins. *Cell* 2000, 102:245–255.

32. Briza P, Eckerstorfer M, Breitenbach M: The sporulation-specific enzymes encoded by the DIT1 and DIT2 genes catalyze a two-step reaction leading to a soluble LL-dityrosine-containing precursor of the yeast spore wall. *Proc Natl Acad Sci U S A* 1994, 91:4524–4528.

33. Engebrecht J, Masse S, Davis L, Rose K, Kessel T: Yeast Meiotic Mutants Proficient for the Induction of Ectopic Recombination. *Genetics* 1998, 148:581–598.

34. Nakayama N, Kaziro Y, Arai K, Matsumoto K: Role of STE genes in the mating factor signaling pathway mediated by GPA1 in Saccharomyces cerevisiae. *Mol Cell Biol* 1988, 8:3777–3783.

35. Zhu YO, Siegal ML, Hall DW, Petrov DA: Precise estimates of mutation rate and spectrum in yeast. *Proc Natl Acad Sci* 2014, 111:E2310–E2318.

36. Lang GI, Murray AW: Estimating the Per-Base-Pair Mutation Rate in the Yeast Saccharomyces cerevisiae. *Genetics* 2008, 178:67–82.

37. Hull CM, Raisner RM, Johnson AD: Evidence for mating of the “asexual” yeast Candida albicans in a mammalian host. *Science* 2000, 289:307–310.

38. Paoletti M, Rydholm C, Schwier EU, Anderson MJ, Szakacs G, Lutzoni F, Debeaupuis J-P, Latgé J-P, Denning DW, Dyer PS: Evidence for sexuality in the opportunistic fungal pathogen Aspergillus fumigatus. *Curr Biol CB* 2005, 15:1242–1248.

39. Muller H, Hennequin C, Gallaud J, Dujon B, Fairhead C: The Asexual Yeast Candida glabrata Maintains Distinct a and□? Haploid Mating Types. *Eukaryot Cell* 2008, 7:848–858.

40. Wong S, Fares MA, Zimmermann W, Butler G, Wolfe KH: Evidence from comparative genomics for a complete sexual cycle in the “asexual” pathogenic yeast Candida glabrata. *Genome Biol* 2003, 4:R10.

41. Gilichinskiy DA, Khlebnikova GM, Zvyagintsev DG, Fedorov-Davydov DG, Kudryavtseva NN: Microbiology of Sedimentary Materials in the Permafrost Zone. *Int Geol Rev* 1989, 31:847–858.

42. Gilichinsky DA, Wilson GS, Friedmann EI, McKay CP, Sletten RS, Rivkina EM, Vishnivetskaya TA, Erokhina LG, Ivanushkina NE, Kochkina GA, Shcherbakova VA, Soina VS, Spirina EV, Vorobyova EA, Fyodorov-Davydov DG, Hallet B, Ozerskaya SM, Sorokovikov VA, Laurinavichyus KS, Shatilovich AV, Chanton JP, Ostroumov VE, Tiedje JM: Microbial populations in Antarctic permafrost: biodiversity, state, age, and implication for astrobiology. *Astrobiology* 2007, 7:275–311.

43. Kochkina GA, Ivanushkina NE, Karasev SG, Gavrish EI, Gurina LV, Evtushenko LI, Spirina EV, Vorob’eva EA, Gilichinskiĭ DA, Ozerskaia SM: [Micromycetes and actinobacteria under conditions of many years of natural cryopreservation]. *Mikrobiologiia* 2001, 70:412–420.

44. Doyle J, Doyle J: A rapid DNA isolation procedure for small quantities of fresh leaf tissue. *Phytochem Bull* 1987, 19:11–15.

45. Luo R, Liu B, Xie Y, Li Z, Huang W, Yuan J, He G, Chen Y, Pan Q, Liu Y, Tang J, Wu G, Zhang H, Shi Y, Liu Y, Yu C, Wang B, Lu Y, Han C, Cheung DW, Yiu S-M, Peng S, Xiaoqian Z, Liu G, Liao X, Li Y, Yang H, Wang J, Lam T-W, Wang J: SOAPdenovo2: an empirically improved memory-efficient short-read de novo assembler. *GigaScience* 2012, 1:18.

46. Schroeder A, Mueller O, Stocker S, Salowsky R, Leiber M, Gassmann M, Lightfoot S, Menzel W, Granzow M, Ragg T: The RIN: an RNA integrity number for assigning integrity values to RNA measurements. *BMC Mol Biol* 2006, 7:3.

47. Kim D, Pertea G, Trapnell C, Pimentel H, Kelley R, Salzberg SL: TopHat2: accurate alignment of transcriptomes in the presence of insertions, deletions and gene fusions. *Genome Biol* 2013, 14:R36.

48. Blanchette M, Kent WJ, Riemer C, Elnitski L, Smit AFA, Roskin KM, Baertsch R, Rosenbloom K, Clawson H, Green ED, Haussler D, Miller W: Aligning multiple genomic sequences with the threaded blockset aligner. *Genome Res* 2004, 14:708–715.

49. Kishino H, Hasegawa M: Evaluation of the maximum likelihood estimate of the evolutionary tree topologies from DNA sequence data, and the branching order in hominoidea. *J Mol Evol* 1989, 29:170–179.

50. Li H, Durbin R: Fast and accurate short read alignment with Burrows-Wheeler transform. *Bioinforma Oxf Engl* 2009, 25:1754–1760.

51. Ranwez V, Harispe S, Delsuc F, Douzery EJP: MACSE: Multiple Alignment of Coding SEquences Accounting for Frameshifts and Stop Codons. *PLoS ONE* 2011, 6:e22594.

52. Huson DH, Richter DC, Rausch C, Dezulian T, Franz M, Rupp R: Dendroscope: An interactive viewer for large phylogenetic trees. *BMC Bioinformatics* 2007, 8:460.

Figure legends

Figure 1. Phylogenetic trees for *P. pannorum* strains. (A) Distances calculated from synonymous sites. Numbers of pseudogenezied or deleted genes are depicted on each branch. (B) Distances calculated from non-synonymous sites. Strains extracted from permafrost are marked with (*). This topology was observed in all 1000 bootstrap iterations.

Figure 2. Genome synteny across *P. pannorum* strains. Each square corresponds to fraction of adjacent gene pairs in strain from vertical row with orthrologs in strain from horizontal row, which are also adjacent in strain from horizontal row.

Figure 3. ((X,Y),Z)/((X,Z),Y)) is a ratio of the number of sites with phylogenetic configuration ((X,Y),Z) to the number of sites with phylogenetic configuration ((X,Z),Y) at distance l to a site with phylogenetic configuration ((X,Y),Z). (A) presents the ratio for strains from the same clade (VKM F-3557, VKM F-4514, VKM F-3808), rooted by VKM F-4246. (B) and (C) presents the ratio for strains from different clades (VKM F-3557, VKM F-4515, VKM F-4246), rooted by VKM F-4519, for synonymous (B) and nonsynonymous (C) sites.

Fig. 4. Genetic structure of the MAT-locus and its flanking regions in *P. spp.* (A) MAT-1 configuration consists of *MAT1-1-3* HMG transcription factor, an unknown gene *MAT1-1-6,* and *MAT1-1-1* α-box transcription factor. The part of the MAT-1 locus which corresponds to region with noncanonical phylogeny is marked with red bar. (B) MAT-2 configuration consists of *MAT1-2-1* HMG-box transcription factor and an unknown gene *MAT1-2-5*. The part of the MAT-2 locus which corresponds to region with noncanonical phylogeny is marked with blue bar. MAT loci are flanked by conservative genes *apn2* and *sla2* in all sequenced strains.

Fig. 5. Phylogenetic configuration of MAT-locus (A) compared to phylogenetic configuration of the region with no recombination (B). Strains which carry both MAT-2 and MAT-1 gene are marked in red, strains which carry MAT-1 gene only are marked in blue. Bootstrap values calculated from 1000 bootstrap iterations.

Fig. 6.. Characteristics of genome regions with noncanonical topologies (VKM F-3557; (VKM F-3808; VKM F-4514)) and (VKM F-4514; (VKM F-3808; VKM F-3557)). (A) Distribution of the regions by their length. (B) Distribution of regions by nucleotide divergence between VKM F-3557 and VKM F-4514.

Fig. 7. An example of the genome region with altered phylogeny across *P.spp*. Multiple sequence alignments of 7 sequenced strains and phylogenetic trees corresponding to them are shown for the region of recombination (B) and for flanking regions (A,C) respectively. Nucleotide sites with canonical topology (VKM F-3808; (VKM F-3557; VKM F-4514)) are shown in green, nucleotide sites with noncanonical topology (VKM F-4514; (VKM F-3557; VKM F-3808)) are shown in red. Bootstrap values for phylogenetic trees were calculated in 1000 replications. This recombination region corresponds to locus #1 in Table S3.

Fig. 8. Evolutionary scenario which explains the observed alignment. Strains F-3808, F-3557, and F-4514 from the same clade are shown as red opposed to all other strains shown as blue. The inverted segment is marked with arrow. Region with noncanonical phylogenetic configuration is marked with black lines. The observed alignment has (VKM F-3557, (VKM F-3808, VKM F-4514)) configuration inside recombination region (marked with short black lines) and (VKM F-3808, (VKM F-3557, VKM F-4514)) canonical configuration in flanking regions. This recombination region corresponds to locus #77(inversion) in Table S3.

Table 1. Habitats and geography of G. pannorum

Strain number (VKM)	Habitat	Geography
F-3808	Temperate environment	Russia, Tverskaya oblast
F-3557	Temperate environment	Sweeden
F-3775	Temperate environment	Germany
F-4246	Temperate environment	Mongolia, Selenge Aimag
F-4281	Cryopeg	Russia, Yakutia, Kolyma lowland
F- 4513	Permafrost	Russia, Yakutia, Kolyma lowland
F-4514	Permafrost	Russia, Yakutia, Kolyma lowland
F-4515	Permafrost	Russia, Yakutia, Kolyma lowland
F-4516	Permafrost	Russia, Yakutia, Kolyma lowland
F-4517	Permafrost	Russia, Yakutia, Kolyma lowland
F-103	Temperate environment	USA, New York
F-4518	Arctic active layer	Russia, Yakutia, Kolyma lowland
F-4519	Arctic active layer	Russia, Yakutia, Kolyma lowland
F-4520	Arctic active layer	Russia, Yakutia, Kolyma lowland

Table 2. Assembly statistics

Strain number (VKM)	Number of reads	Coverage	Assembly length	Average contig length	Longest contig length	N50
F-3808	23,424,660	27	31,376,466	12,801	126,211	21,839
F-3557	10,744,922	11	26,960,732	11,950	128,114	24,755
F-3775	9,492,087	9	26,619,547	5,672	67,045	9,307
F-4246	8,947,406	9	24,833,625	10,531	132,394	22,823
F-4281	27,370,574	25	23,704,604	10,355	105,778	21,424
F-4513	18,238,108	20	24,207,568	12,794	135,020	28,067
F-4514	21,533,593	21	24,946,410	15,456	143,204	30,182
F-4515	27,051,031	15	30,802,195	16,560	204,738	39,825
F-4516	26,615,833	32	25,236,587	11,909	202,070	63,620
F-4517	26,789,498	28	31,131,070	6,388	157,401	22,962
F-103	20,880,571	23	27,749,379	25,982	209,525	55,172
F-4518	17,007,142	15	30,987,437	11,858	183,039	30,119
F-4519	16,072,124	15	28,406,515	12,744	176,316	27,918
F-4520	14,193,026	12	29,758,268	9,444	138,716	22,176

Table 3. The key parameters of annotated genomes of P. spp.

Strain number (VKM)	GC-content	Number of genes	Average gene length (bp)	Number of introns per gene	Average intron length (bp)	Median intron length (bp)	CEGMA complete	CEGMA partial
F-3808	50.54%	11,305	1647	2.19	108	60	92%	97%
F-3557	50.23%	10,717	1677	2.12	106	59	90%	96%
F-3775	49.08%	11,592	1448	1.78	102	58	62%	72%
F-4246	51.07%	9,516	1724	1.99	103	58	90%	98%
F-4281	50.52%	9,593	1727	2.01	108	59	94%	98%
F- 4513	50.86%	9,605	1747	2.01	103	58	95%	99%
F-4514	50.50%	10,277	1747	2.24	108	60	96%	98%
F-4515	50.17%	11,636	1783	2.48	111	59	96%	99%
F-4516	49.93%	10,125	1799	2.21	105	59	98%	100%
F-4517	49.97%	11,972	1629	1.97	104	59	96%	98%
F-103	50.31%	10,441	1828	2.20	106	59	97%	99%
F-4518	50.02%	12,470	1752	2.15	109	59	96%	98%
F-4519	50.12%	11,466	1752	2.11	108	59	96%	99%
F-4520	50.26%	12,612	1697	2.08	107	59	96%	98%

Table 4.

Strains forming a clade	Number of sites in aligment centerd on a clade (nt)	Support a clade (nt)	Conflict with a clade (nt)
(VKM F-3808, VKM F-3557, VKM F-4514, VKM F-4516)	17,307,123	16,652,769	81,744
(VKM F-4246, VKM F-4513)	15,132,092	14,901,548	46,377
(VKM F-4515, VKM F-4517)	15,268,980	15,220,882	8,318
(VKM F-103, VKM F-4519)	14,941,621	13,805,217	189,664
(VKM F-4518, VKM F-4520)	15,382,763	14,838,267	88,539

Table 5. Meiotic genes and mating pathway genes in P. pannorum

**gene in S. cerevisiae**	**ortholog in P. pannorum**	**Function in S. cerevisiae**
csm1	+	chromosome segregation
csm3	+	chromosome segregation
dit1	-	pyoverdine/dityrosine biosynthesis
gsg1	+	late post-replication meotic role
hop2	+	prevents synapsis between non-homologous chromosomes
ime1	-	meiosis-inducing protein 1
ime2	+	kinase, stimulates meiotic gene expression
isc10	-	sporulation
mck1	+	kinase required for ime1 expression
mek1	+	a protein kinase that displays genetic interactions with RED1 and HOP1
mnd1	+	recombination and meiotic nuclear division
msh4	+	required for cross-over during meiosis
msh5	+	MutS homolog, facilitates meiotic reciprocal recombination between homologs
mum2	-	required for premeiotic DNA synthesis and sporulation
mum3	-	required for premeiotic DNA synthesis and sporulation
rad1	+	DNA repair protein
rad17	+	DNA replication and repair
rec12	-	meiosis induction
red1	-	gene required for meiosis I
rim4	-	activation of sporulation-specific genes
smk1	+	sporulation specific MAP-kinase
spo1	+	spindle body duplication
spo11	+	meiosis initiation by formation of double-strand breaks in DNA
spo14	+	commitment to meiosis
spo22	-	chromosome segregation
spo75	+	required for spore and ascus formation
ume6	-	regulator of early meiotic gene expression, DNA binding protein
zip1	-	meiotic chromosome synapse
zip2	-	meiotic synaptoname complex
zip3	-	meiotic synaptoname complex
zip4	-	meiotic synaptoname complex
akr1	+	required for endocytosis of pheromone receptors
cdc24	+	required for polarity establishment and maintenance; mutants have morphological defects in bud formation and shmooing
cdc42	+	essential for establishment and maintenance of cell polarity
far1	+	inhibitor of Cdc28-Cln complex
fus3	+	activates Ste12 and Far1

lsg1	+	required for mating and sporulation
opy2	+	overproduction blocks cell cycle arrest in the presence of mating pheromone
pea2	-	required for phermone-induced pointed projection formation
sgv1	+	pheromone adaptation
spa2	+	pheromone-induced morphogenesis and efficient mating.
ste2	+	pheromone mating factor
ste3	+	pheromone A receptor
ste4	+	pheromone signal transduction
ste6	+	ABC-type multidrug transport system
ste7	+	pheromone signal transduction
ste11	+	Ser/Thr protein kinase; pheromone signal transduction
ste12	+	activates genes involved in mating or pseudohyphal/invasive growth pathways
ste13	+	peptidase,mating factor processing
ste18	+	signal transduction via G-protein-coupled receptors
ste20	+	activates transcription of FUS1 in the absence of mating pheromone
ste50	+	essential for activation of conjugation

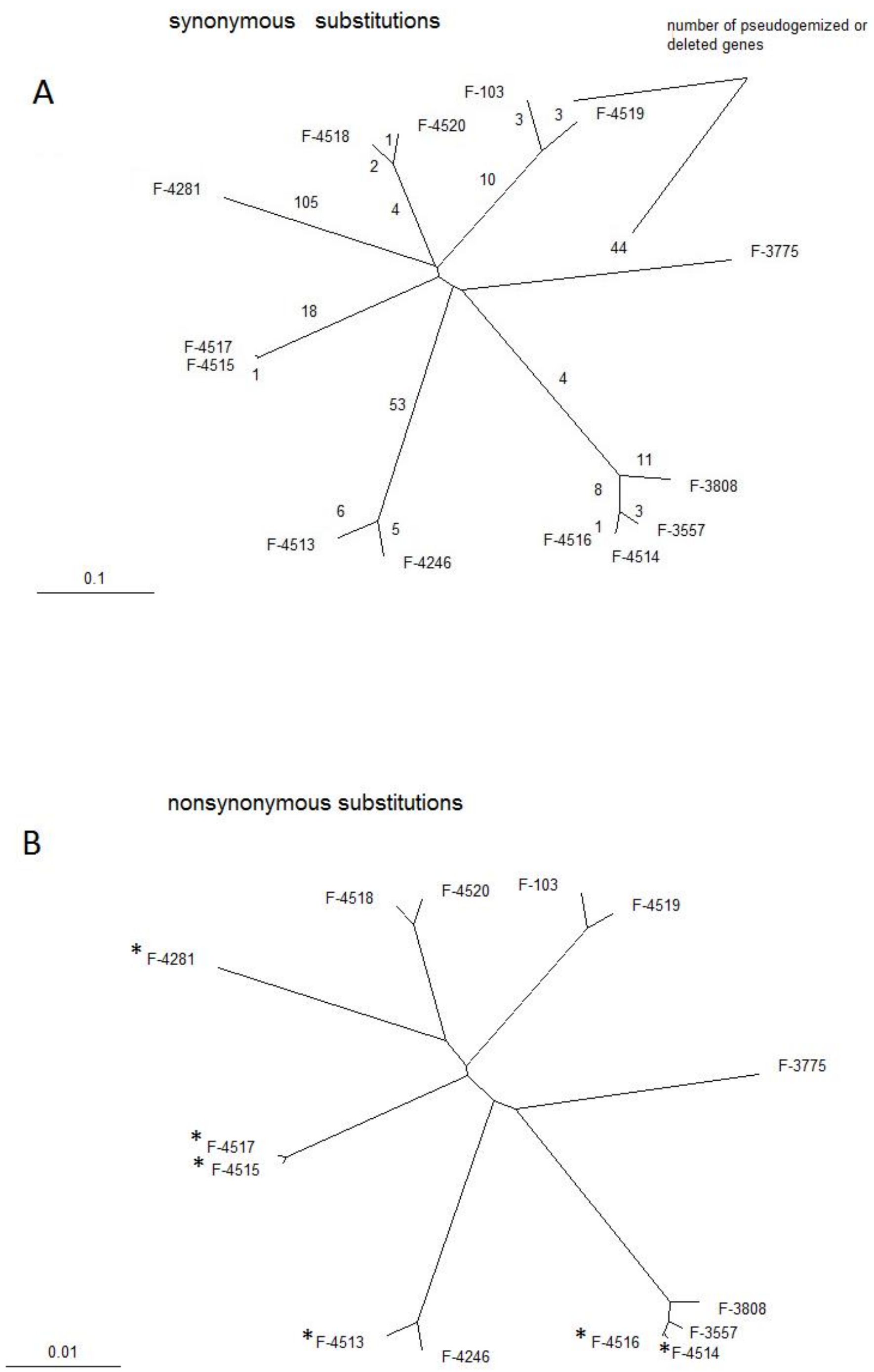
synonymous substitutions
number of pseudogemized or deleted genes
A
F-103
3
3
F-4519
F-4520
F-4518
1
2
10
F-4281
105
4
44
F-3775
18
F-4517
F-4515
1
4
53
11
F-3808
8
3
F-3557
6
5
1
F-4513
F-4516
F-4514
F-4246
0.1
nonsynonymous substitutions
B
F-4518
F-4520
F-103
F-4519
*F-4281
F-3775
*F-4517
*F-4515
F-3808
F-3557
*F-4513
*F-4516
*F-4514
F-4246
0.01

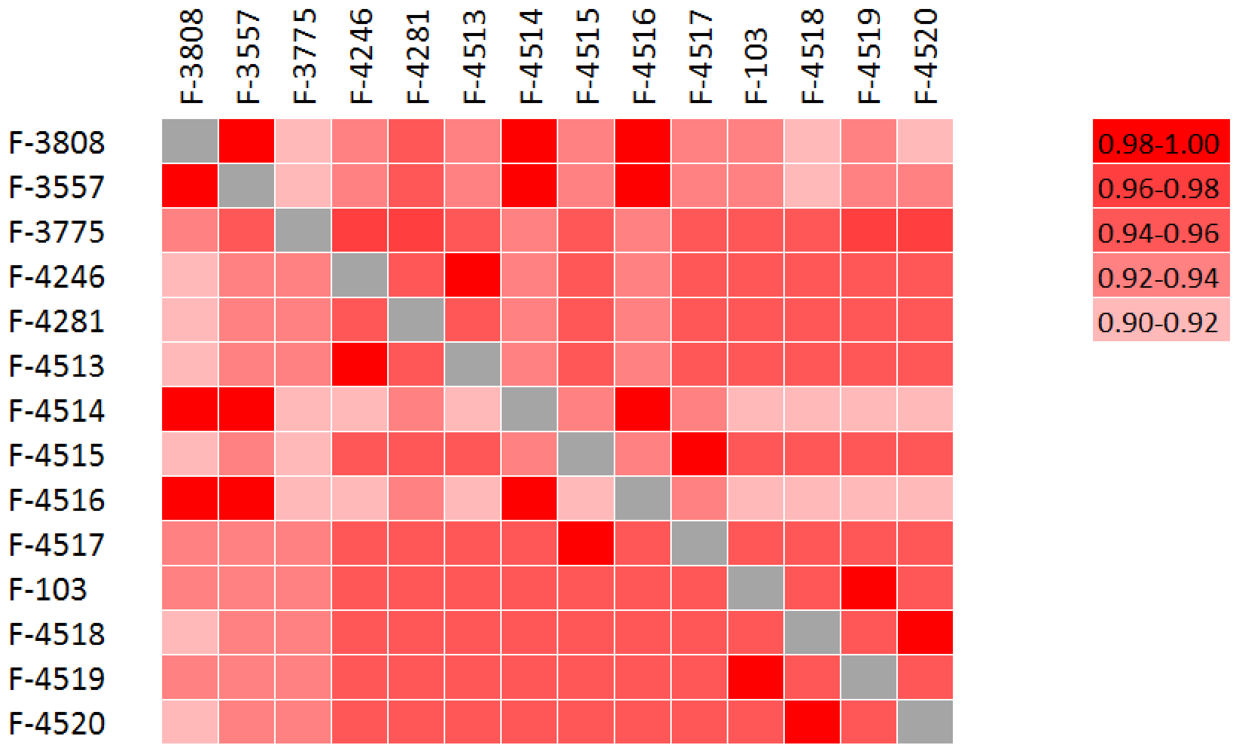
F-3808
F-3557
F-3775
F-4246
F-4281
F-4513
F-4514
F-4515
F-4516
F-4517
F-103
F-4518
F-4519
F-4520
0.98-1.00
0.96-0.98
0.94-0.96
0.92-0.94
0.90-0.92

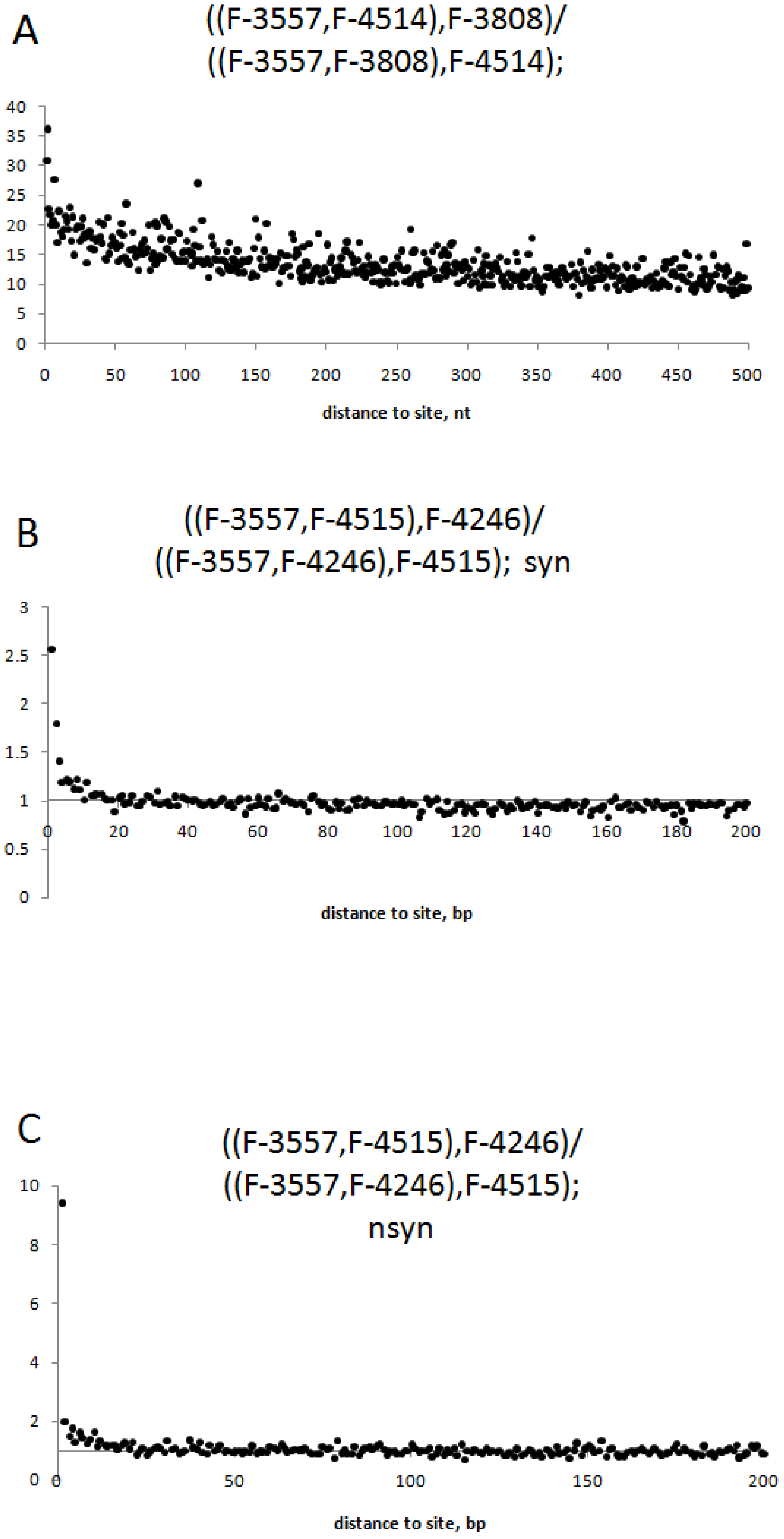
A
((F-3557,F-4514),F-3808)/
((F-3557,F-3808),F-4514);
40
35
30
25
20
15
10
5
0
0 50 100 150 200 250 300 350 400 450 500
distance to site, nt
B
((F-3557,F-4515),F-4246)/
((F-3557,F-4246),F-4515); syn
3
2.5
2
1.5
1
0.5
0
0 20 40 60 80 100 120 140 160 180 200
distance to site, bp
C
((F-3557,F-4515),F-4246)/
((F-3557,F-4246),F-4515);
nsyn
10
8
6
4
2
0
0 50 100 150 200
distance to site, bp

644

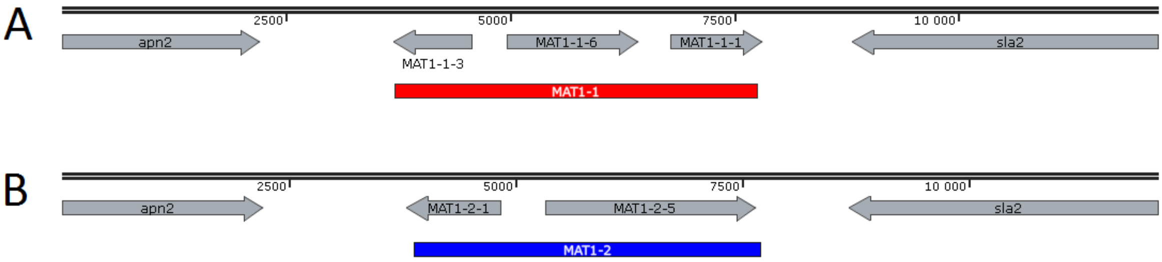
A
2500
5000
7500
10 000
apn2
MAT1-1-3
MAT1-1-6
MAT1-1-1
sla2
MAT1-1
B
2500
5000
7500
10 000
apn2
MAT1-2-1
MAT1-2-5
sla2
MAT1-2

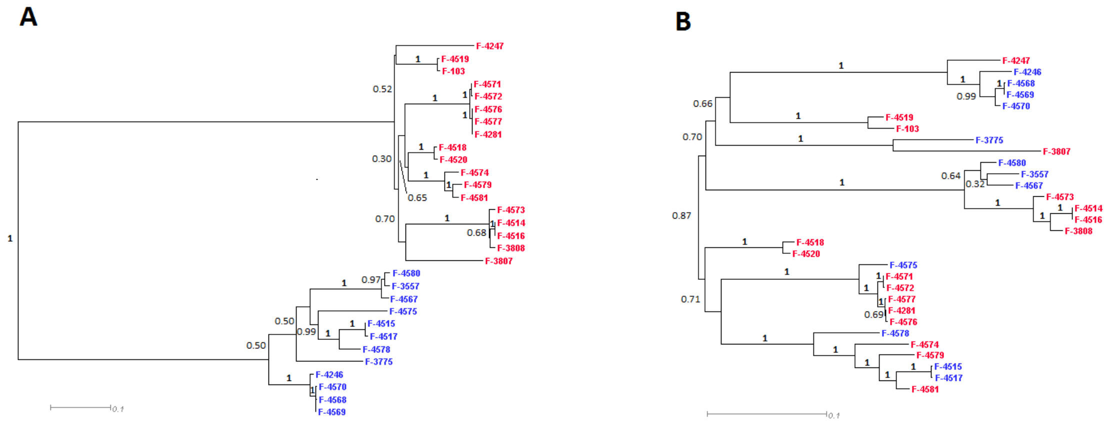
A
F-4247
F-4519
F-103
F-4571
F-4572
F-4576
F-4577
F-4281
F-4518
F-4520
F-4574
F-4579
F-4581
F-4573
F-4514
F-4516
F-3808
F-3807
F-4580
F-3557
F-4567
F-4575
F-4515
F-4517
F-4578
F-3775
F-4246
F-4570
F-4568
F-4569
0.1
B
F-4247
F-4246
F-4568
F-4569
F-4570
F-4519
F-103
F-3775
F-3807
F-4580
F-3557
F-4567
F-4573
F-4514
F-4516
F-3808
F-4518
F-4520
F-4575
F-4571
F-4572
F-4577
F-4281
F-4576
F-4578
F-4574
F-4579
F-4515
F-4517
F-4581
0.1

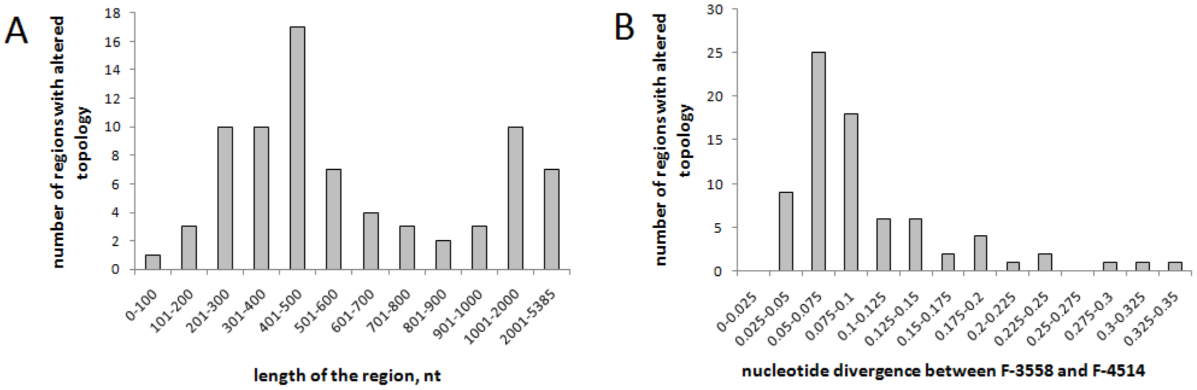
A
number of regions with altered topology
0
2
4
6
8
10
12
14
16
18
0-100
101-200
201-300
301-400
401-500
501-600
601-700
701-800
801-900
901-1000
1001-2000
2001-5385
length of the region, nt
B
number of regions with altered topology
0
5
10
15
20
25
30
0-0.025
0.025-0.05
0.05-0.075
0.075-0.1
0.1-0.125
0.125-0.15
0.15-0.175
0.175-0.2
0.2-0.225
0.225-0.25
0.25-0.275
0.275-0.3
0.3-0.325
0.325-0.35
nucleotide divergence between F-3558 and F-4514

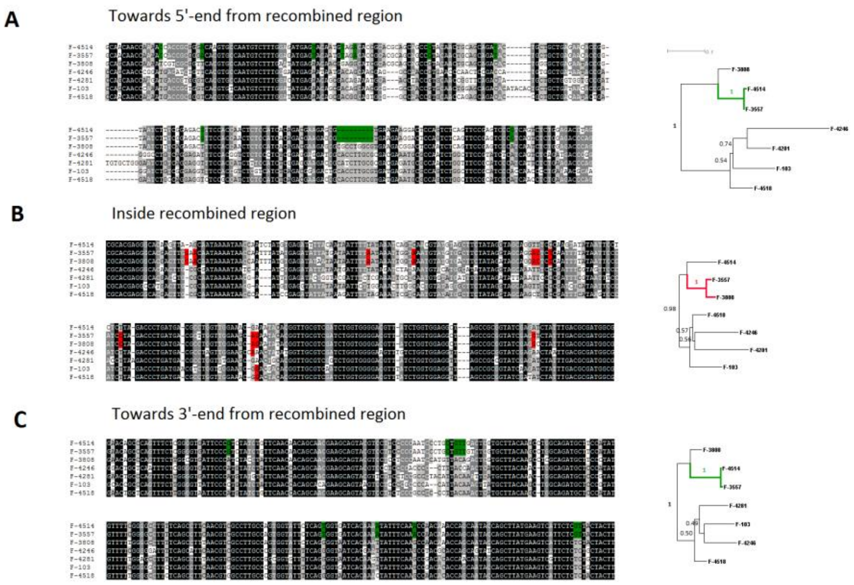
A
Towards 5'-end from recombined region
B
Inside recombined region
C
Towards 3'-end from recombined region

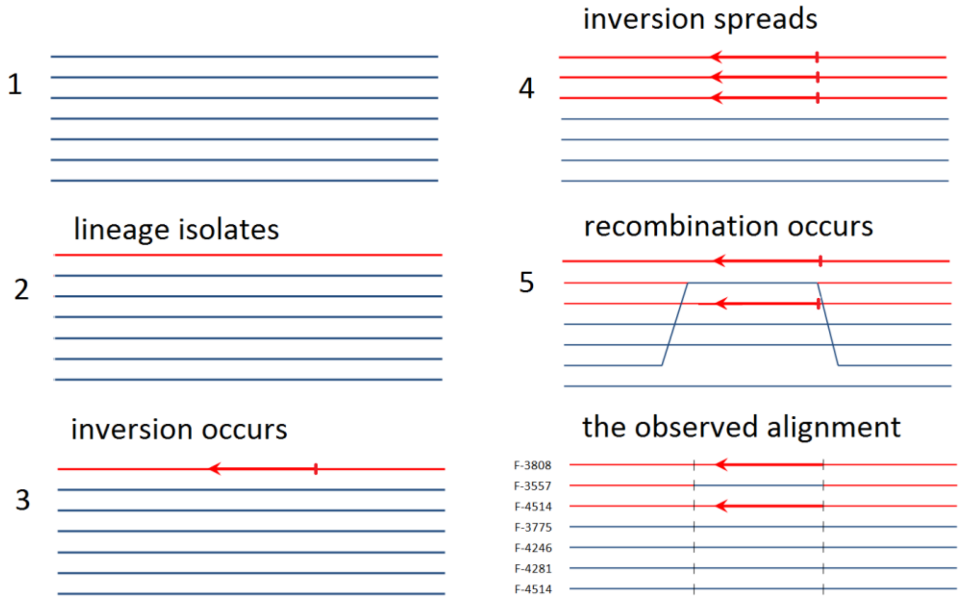
1
lineage isolates
2
inversion occurs
3
inversion spreads
4
recombination occurs
5
the observed alignment
F-3808
F-3557
F-4514
F-3775
F-4246
F-4281
F-4514
